# Synergistic Influence of Yeast Extract and Calcium Oxide Nanoparticles on the Synthesis of Bioactive Antioxidants and Metabolites in *Swertia chirata* In Vitro Callus Cultures

**DOI:** 10.3390/molecules28124607

**Published:** 2023-06-07

**Authors:** Tauqeer Sardar, Mehwish Maqbool, Muhammad Ishtiaq, Muhammad Waqas Mazhar, Mohamed A. El-Sheikh, Ryan Casini, Eman A. Mahmoud, Hosam O. Elansary

**Affiliations:** 1 Department of Botany, Mirpur University of Science and Technology, Mirpur 10250, Pakistan; 2 Botany and Microbiology Department, College of Science, King Saud University, P.O. Box 2460, Riyadh 11451, Saudi Arabia; 3 School of Public Health, University of California, 2121 Berkeley Way, Berkeley, CA 94704, USA; 4 Department of Food Industries, Faculty of Agriculture, Damietta University, Damietta 34511, Egypt; emanmail2005@yahoo.com; 5 Plant Production Department, College of Food and Agriculture Sciences, King Saud University, P.O. Box 2460, Riyadh 11451, Saudi Arabia

**Keywords:** *Swertia chirata*, elicitors, phytochemicals, antioxidant, in vitro culture, CaONPs, wild herbal drugs

## Abstract

The challenges in the production of metabolites of medicinal potential from wild plants include low yields, slow growth rates, seasonal variations, genetic variability and regulatory as well as ethical constraints. Overcoming these challenges is of paramount significance and interdisciplinary approaches and innovative strategies are prevalently applied to optimize phytoconstituents’ production, enhance yield, biomass, ensure sustainable consistency and scalability. In this study, we investigated the effects of elicitation with yeast extract and calcium oxide nanoparticles (CaONPs) on in vitro cultures of *Swertia chirata* (Roxb. ex Fleming) Karsten. Specifically, we examined the effects of different concentrations of CaONPs in combination with different concentrations of yeast extract on various parameters related to callus growth, antioxidant activity, biomass and phytochemical contents. Our results showed that elicitation with yeast extract and CaONPs had significant effects on the growth and characteristics of callus cultures of *S. chirata*. The treatments involving yeast extract and CaONPs were found to be the most effective in increasing the contents of total flavonoid contents (TFC), total phenolic contents (TPC), amarogentin and mangiferin. These treatments also led to an improvement in the contents of total anthocyanin and alpha tocopherols. Additionally, the DPPH scavenging activity was significantly increased in the treated samples. Furthermore, the treatments involving elicitation with yeast extract and CaONPs also led to significant improvements in callus growth and characteristics. These treatments promoted callus response from an average to an excellent level and improved the color and nature of the callus from yellow to yellow-brown and greenish and from fragile to compact, respectively. The best response was observed in treatments involving 0.20 g/L yeast extract and 90 ug/L CaONPs. Overall, our findings suggest that elicitation with yeast extract and CaONPs can be a useful strategy for promoting the growth, biomass, phytochemical contents and antioxidant activity of callus cultures of *S. chirata* in comparison to wild plant herbal drug samples.

## 1. Introduction

The wild growing plant *Swertia chirata* (Roxb. ex Fleming) Karsten, also known as Chirata or gentian herb, is a medicinal plant species that is native to the Himalayan region of India, Nepal, Pakistan and Bhutan. It is a member of the Gentianaceae family and is widely used in traditional Ayurvedic medicine for its bitter and cooling properties [1]. *Swertia chirata* is valued for its medicinal properties and has been traditionally used to treat a variety of ailments, including fever, liver disorders, digestive issues, skin infirmities and as anti-aging. It is believed to have anti-inflammatory, anti-microbial, anti-cancer and immune-modulating properties. Research has shown that Swertia *chirata* contains a variety of bioactive compounds, including xanthones, iridoids, phenols and flavonoids, which are responsible for its medicinal properties. These compounds have been found to exhibit antioxidant, anti-inflammatory, anti-aging and anti-microbial activities, as well as potential anti-cancer effects [2]. *Swertia chirata* is commonly consumed as a tea or extract [3].

Currently, the major issue is the scarce population of *Swertia chirata* due to over-exploitation and habitat loss. Secondly, adulteration makes it more ambiguous and unsafe for its use as botanic drug in local communities. To overcome this plethora of issues and obtain consistent and reliable phytoconstituents in reasonable amounts, the in vitro culturing approach was employed. In latest techniques, in vitro callus cultures are widely used for the production of highly valuable botanic drugs, which provides a high concentration of plant secondary metabolites such as alkaloids, phenolics, flavonoids and terpenoids which are the key source of cure of various infirmities.

The technique of callus cultures is a mass of undifferentiated cells that can be induced to produce secondary metabolites under controlled conditions [4]. The use of callus cultures for the production of botanic drugs offers several advantages over traditional methods of plant cultivation, growth and subsequent extraction of phytoconstituents. For example, callus cultures can be maintained in a controlled environment, allowing for the production of high-quality, consistent plant material regardless of season or geographic location. Additionally, the use of callus cultures can reduce the need for wild harvesting of rare or endangered plant species alleviating biotic threats for plants to become extinct [5].

The production of botanicals from callus cultures typically involves inducing the callus to differentiate into a specific tissue type, such as roots or shoots, and then stimulating the tissue to produce the desired secondary metabolites [6]. This can be achieved through various methods, such as altering the culture medium or applying elicitors such as jasmonic acid or salicylic acid [7,8].

Antioxidant metabolites are a collection of compounds that can safeguard cells against damage caused by oxidative stress [9]. Oxidative stress arises when there is an imbalance between reactive oxygen species (ROS) production and the cell’s ability to eliminate or repair the resulting damage [10]. This kind of stress can result in cellular damage, which is connected with a range of diseases such as neurodegenerative disorders, cancer, aging and cardiovascular disease. These metabolites are believed to exhibit antioxidant properties by neutralizing free radicals and reactive oxygen species, thus minimizing oxidative damage to cells [11]. A large number of plant-based foods such as whole grains, vegetables and fruits are rich sources of antioxidant metabolites. Eating a diet that is high in antioxidant-rich foods has been connected with a reduced risk of chronic diseases such as cancer, cardiovascular disease, aging and diabetes. In addition to their possible health benefits, antioxidant metabolites are also used as natural preservatives and colorants in the food and cosmetic industries. These metabolites can extend the shelf life of food products by preventing lipid oxidation and improving the stability of cosmetic formulations [12].

Amarogentin, mangiferin and swertiamarin are three bioactive compounds found in plants that have been studied for their potential health benefits as key potential drugs [13]. Amarogentin is a bitter compound found in the roots of *Swertia chirata*, a plant species native to the Himalayan region of India and Palandri area of Azad Jammu and Kashmir, Pakistan. It has been shown to have anti-inflammatory, anti-microbial, anti-aging and anti-cancer properties. Amarogentin is believed to work by inhibiting the activity of inflammatory enzymes and promoting apoptosis (cell death) in cancer cells [14]. Mangiferin is a natural xanthone compound found in mangoes and other plant species, such as Anemarrhena asphodeloides and Salacia reticulata. It has been shown to have antioxidant, anti-inflammatory, anti-wrinkle and anti-diabetic properties. Mangiferin is believed to work by reducing oxidative stress and inflammation in the body as well as regulating glucose metabolism. Similarly, swertiamarin is believed to work by reducing oxidative stress and inflammation in the body, as well as promoting the regeneration of liver cells and facial skin [14,15].

Calcium oxide nanoparticles (CaONPs) have been studied as potential elicitors in plants. Elicitors are substances that can trigger a defense response in plants, leading to increased resistance against pathogens or pests [16]. Studies have shown that CaONPs can induce various defense responses in plants [17], such as the production of reactive oxygen species, the activation of defense-related enzymes and the accumulation of phytohormones. These responses can enhance the plant’s ability to resist biotic and abiotic stresses. Furthermore, CaONPs have been found to enhance the production of secondary metabolites in plants, such as phenolics and flavonoids, which are known for their antioxidant and anti-inflammatory properties [18].

Yeast extracts are commonly used as elicitors in plant cell and tissue culture systems to stimulate the production of secondary metabolites which are the main source of drug discovery and development [19]. Yeast extracts are a rich source of carbohydrates, amino acids, vitamins and minerals, and they contain a variety of bioactive molecules, including β-glucans, mannan oligosaccharides and nucleotides [20]. These bioactive molecules are believed to act as elicitors by binding to specific receptors on the plant cell surface, triggering a cascade of signaling events that lead to the production of secondary metabolites. Research has shown that yeast extracts can increase the production of secondary metabolites in a range of plant species, including medicinal plants, vegetables and fruits. The use of yeast extracts as elicitors can also improve the nutritional quality of plant products by increasing the concentration of antioxidants and other bioactive compounds [21]. One advantage of using yeast extracts as elicitors is that they are relatively inexpensive and readily available, making them a practical option for large-scale plant cell and tissue culture systems. Additionally, yeast extracts are considered safe for use in food and pharmaceutical products and they do not pose significant environmental risks [19,20,21].

The study aimed to investigate how yeast extracts and calcium oxide nanoparticles interact to affect the production of secondary metabolites in *Swertia chirata* callus cultures. The specific objectives were to determine the optimal concentrations of yeast extracts and calcium oxide nanoparticles for maximizing secondary metabolite production in the callus cultures. Additionally, the study aimed to assess the effects of different combinations of yeast extracts and calcium oxide nanoparticles on the production of specific secondary metabolites and understand the underlying mechanisms involved. Furthermore, the potential for scaling up secondary metabolite production in the callus cultures using the optimized treatment with yeast extracts and calcium oxide nanoparticles was also evaluated.

## 2. Results

### 2.1. Impact on Callus Nature Response and Color

The treatments involving elicitation with yeast extract and CaONPs showed significant effects on the growth and characteristics of callus cultures of *Swertia chirata* (Table 1). Specifically, these treatments were found to promote callus response from an average to an excellent level. The color of the callus was also improved from yellow to yellow-brown and greenish, with the best response observed in treatments T_14_, T_15_ and T_16_. In addition, the nature of the callus was improved from fragile to compact in these treatments. In detail, the best callus responses were observed in treatments T_14_, T_15_ and T_16_. which involved elicitation with yeast extract and CaONPs. Specifically, T_16_, which used 0.20 g/L yeast extract and 90 μg/L CaONPs, showed the most pronounced effects on callus growth and characteristics, with a significant improvement in callus response, color and nature compared to the control treatment (T_1_). In T_16_, the callus response improved from an average to an excellent level, and the color improved from yellow to yellow-brown and greenish. The nature of the callus also changed from fragile to compact, indicating a more robust and healthy growth pattern (Table 1). Treatments T_14_ and T_15_ also showed significant improvements in callus growth and characteristics, although to a lesser extent than T_16_. Specifically, these treatments led to an improvement in callus response. Overall, these findings suggest that elicitation with yeast extract and CaONPs can be a useful strategy for promoting the growth and characteristics of callus cultures of *Swertia chirata*, leading to a more robust and healthy growth pattern.

### 2.2. Impact of Elicitation on Callus Growth and Biomass

In addition to improving the callus nature and response, the treatments involving elicitation with yeast extract and CaONPs also showed significant effects on various parameters related to plant growth in the in vitro cultures of *Swertia chirata*. Treatments T_14_, T_15_ and T_16_ were found to be the most effective in terms of improving shoot length, root length, number of shoots per explant and number of roots per shoot ratios. Specifically, T_16_, which involved elicitation with 0.20 g/L yeast extract and 90 ug/L CaONPs, showed the most pronounced effects on all of these parameters, with significant increases observed compared to the control treatment (T_1_). T_14_ and T_15_ also showed significant improvements in these parameters, although to a lesser extent than T_16_. Overall, these findings suggest that elicitation with yeast extract and CaONPs can be a useful strategy for enhancing the growth and development of *Swertia chirata* cultures in addition to improving the production of bioactive compounds. It is worth noting, however, that the optimal concentrations of yeast extract and CaONPs may depend on the specific parameters being targeted, and further optimization studies may be needed to maximize the benefits of these elicitors (Table 2).

### 2.3. Efficacy of Elicitors in Improving Callus Moisture Contents, DPPH Scavenging Percentage, Tocopherols and Anthocyanin Values

Our study showed that the treatments involving elicitation with yeast extract and CaONPs led to significant improvements in various parameters related to the in vitro cultures of *Swertia chirata*. Specifically, T_14_, T_15_ and T_16_ were found to be the most effective treatments in terms of improving callus moisture contents, DPPH scavenging percentage, total anthocyanin and alpha tocopherols. T_16_, which involved elicitation with 0.20 g/L yeast extract and 90 ug/L CaONPs, led to significant increases in callus moisture contents, DPPH scavenging percentage, total anthocyanin and alpha tocopherols most effectively among all treatments compared to the control treatment (T_1_). T_15_ and T_16_ also showed significant improvements in these parameters, with T_16_ having the most pronounced effects. It is worth noting that the individual effects of the different treatments on these parameters were not as significant as the combined effects. This suggests that the use of yeast extract and CaONPs together can be a more effective strategy for improving the production of bioactive compounds in *Swertia chirata* cultures compared to using these elicitors individually. Overall, our findings highlight the potential of elicitation with yeast extract and CaONPs as a tool for enhancing the nutritional and medicinal properties of *Swertia chirata* (Table 3).

### 2.4. Effect of Callus Elicitation on Production of Amarogentin, Mangiferin, Total Flavonoids and Total Phenolics

The data presented in Table 4 show the effect of elicitation on different useful bioactive compounds of *S. chirata*. Among the different treatments tested, T_14_, T_15_ and T_16_ showed the most significant effects on the in vitro cultures of *Swertia chirata*. These treatments, which involved elicitation with 0.20 g/L yeast extract and 30, 60 and 90 μg/L CaONPs, respectively, led to the highest increases in the contents of total flavonoids, total phenolics, amarogentin and mangiferin. Specifically, T_16_ increased the contents of total flavonoids and total phenolics, while also leading to significant increases in the levels of amarogentin and mangiferin. T_15_ and T_16_ also showed significant increases in the contents of total flavonoids, total phenolics, amarogentin and mangiferin, with T_16_ having the most pronounced effects. Overall, our findings suggest that elicitation with yeast extract and CaONPs can be a useful strategy for enhancing the production of bioactive compounds in *Swertia chirata* cultures. The LSD test results are presented in the table, which shows that means with different letters following them, ranked by their significance, have significantly different values.

### 2.5. Elicitation Impact on Bioactive Antioxidants

The data presented in Table 5 demonstrate the effects of elicitation treatments on the in vitro callus cultures of *S. chirata*. Overall, the effect of 16 treatments as per the treatment plan was studied on the activity of antioxidant metabolites. Callus elicitation with all the treatments significantly affected the functions of antioxidants. The LSD test results are presented in the table, which shows that means with different letters following them, ranked by their significance, have significantly different values. The control group not treated with an elicitor exhibited the lower functions of PAL, SOD, POD and CAT. However, elicitation increases the functions of assayed enzymes. The treatment with CaONPs and YE either alone or in combination provided a significant increase in the activities of these enzymes. The best results were achieved with the treatments, i.e.,T_14_, T_15_ and T_16_ where higher doses of YE and CaONPs were used in combination as the elicitation treatment. Callus elicitation with 0.20 g/L YE and 90 μg/L CaONPs caused the maximum increase in the activities of antioxidant metabolites in the in vitro callus cultures of *S. chirata*.

### 2.6. Statistical Assessment on the Studied Variables

**Figure 1:**
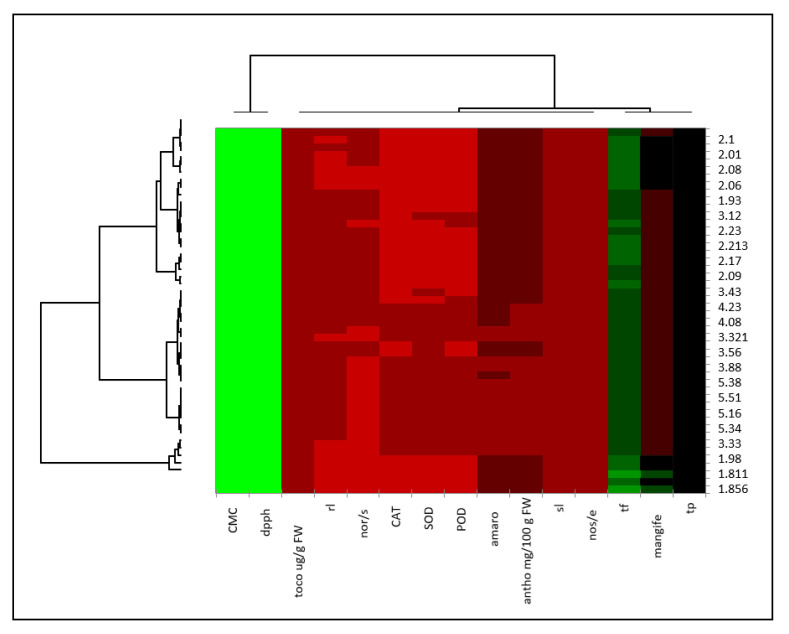
Graphical representation of data using color-coding to display the relative density or intensity of values across a two-dimensional space. The *x* and *y* axes of the heatmap represent the two dimensions of the data being visualized.

In the study, a correlation analysis (Table 6) was conducted to explore the relationships among the variables studied. The analysis revealed that there were positive and significant correlations among the variables. To better visualize these relationships, a heatmap was constructed based on the data. The heatmap provided a graphical representation of the correlations, with different colors indicating the strength and direction of the correlations (Figure 1). This allowed the researchers to easily identify patterns and relationships among the variables, which helped in the interpretation of the results. Overall, the heatmap was a useful tool in understanding the correlations among the variables and helped to strengthen the findings of the study.

In the study, a two-way analysis of variance (ANOVA) was performed to investigate the effect of treatment in rows and columns, as well as their interaction, on the variable of interest (Table 7). The analysis revealed that both the row and column treatments had a significant effect on the variable. Additionally, the interaction between the row and column treatments was also found to be significant. This suggests that the effects of the row and column treatments on the variable were not independent of each other, but rather, they interacted with each other to produce a combined effect. The significant interaction between the treatments indicates that the effect of one treatment may be different depending on the level of the other treatment. Overall, the two-way ANOVA provided valuable information on the effects of the treatments on the variable of interest and helped to identify important interactions between them.

**Figure 2:**
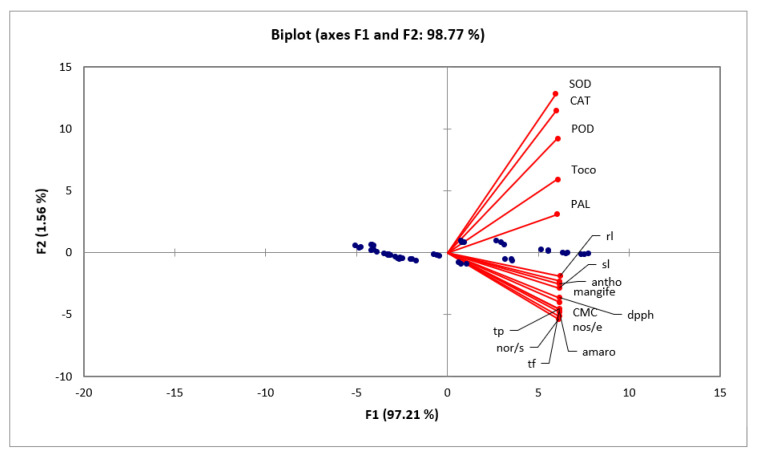
Principal component analysis biplot showing aggregation of variables studied in the positive quarter depicting positive correlation among the studied variables.

PCA loading charts have been shown in Figure 2, which depicts the aggregation of studied variables in positive quarter showing positive and significant correlation among the studied variables.

## 3. Discussion

In the present study, we noticed an enhanced cultivation rate, biomass and growth traits in terms of shoot length, root length, number of shoots per explant and number of roots to shoot ratios. Yeast extracts contain various nutrients, including amino acids, vitamins, and minerals, that can be beneficial for plant growth and development [22]. When added to the plant tissue culture medium, yeast extracts can serve as an effective source of nutrients for the callus culture, promoting cell division and accelerating the rate of growth. Yeast extracts can also contain plant growth regulators, such as auxins and cytokinin, which can stimulate cell division and elongation [23]. Presumably, the presence of these nutrients might have increased biomass production in the in vitro callus cultures. These growth regulators can help in the induction and maintenance of callus growth. Similarly, the application of CaONPs improved callus growth and biomass. In this respect, a study from the literature [24] reported that calcium oxide nanoparticles can enhance nutrient uptake and transport in the callus cultures, which can increase the availability of nutrients for cell growth and division. Nanoparticles can release calcium ions, which can act as signaling molecules and regulate nutrient uptake and metabolism. Additionally, nanoparticles can increase the surface area of the substrate, which can provide more contact between the cells and the nutrient medium, promoting nutrient absorption. It was noticed that callus moisture contents were also improved upon treatment with CaONPs. Studies have shown that calcium oxide nanoparticles improve the water-holding capacity of the callus cultures, which can increase the moisture content and prevent water loss [25,26]. Furthermore, nanoparticles can form a protective layer on the surface of the cells, which can reduce the rate of transpiration and prevent dehydration. Increased moisture contents of the callus cultures might be due to CaONPs. The increased number of shoots per explant and better callus growth might be due to Ca^2+^. The authors of [27] reported that calcium oxide nanoparticles can activate plant growth-promoting pathways in the callus cultures. Nanoparticles can enter the cells and interact with the genetic material, leading to changes in gene expression and activation of growth-promoting pathways. For example, nanoparticles can upregulate the expression of genes involved in cell division and expansion, leading to increased callus growth. It might be assumed from the discussion that CaONPs and yeast extract are potential elicitors to enhance callus growth and production.

The application of CaONPs and yeast extracts improved callus nature and response. Furthermore, the callus color traits were examined and it was seen that elicitation increases callus compactness, greenish color and callus setting. Calcium oxide nanoparticles can improve the response of callus cultures by increasing the production of anthocyanins, which are responsible for the color properties of the cells. Anthocyanins are known to be influenced by many factors, including light, temperature, pH and nutrient availability [28]. In our study, the anthocyanin contents were seen to improve upon elicitation treatments. These findings coincide with our results. Calcium oxide nanoparticles may affect the color properties of callus cultures by altering the availability of nutrients and minerals, such as calcium, that are required for anthocyanin biosynthesis. Additionally, the nanoparticles may act as a signal to the cells, triggering the activation of genes and enzymes involved in anthocyanin biosynthesis [29]. Furthermore, the small size of the nanoparticles may allow them to penetrate into the cells and affect their metabolic pathways, leading to changes in the color properties of the callus cultures. Similarly, the application of yeast extract improved callus nature, texture, response and color. Yeast extract contains various nutrients that can improve callus growth, including the accumulation of secondary metabolites, which can enhance the color and moisture content of callus tissue. Yeast extract can provide essential amino acids and vitamins, which are necessary for cell division and differentiation, leading to healthy callus growth [30]. Moreover, yeast extract contains plant growth regulators, such as auxins and cytokinins, that can stimulate callus formation and growth, leading to the production of healthy and robust callus tissue. The cytokinins present in yeast extract can also enhance the moisture content of callus tissue, leading to better hydration and improved growth. Furthermore, yeast extract can act as an elicitor, which triggers the production of secondary metabolites, including pigments, such as anthocyanins, which can enhance the color of callus tissue [31]. Additionally, the presence of phenolic compounds, such as flavonoids and tannins, can increase the antioxidant capacity of the callus tissue, leading to improved color and texture. Presumably, the presence of nutrients, growth regulators, and elicitors in yeast extract improved the nature, response, color and moisture content of callus tissue [32].

Tocopherols, also known as Vitamin E, are lipid-soluble antioxidants that protect cell membranes and other lipids from oxidative damage. In the present study, the tocopherol contents were seen to be boosted by elicitation treatments. The authors of [33] reported that yeast extract contains various compounds that can stimulate the biosynthesis of tocopherols in plant cells. One of the mechanisms by which yeast extract can increase tocopherol content is through the activation of the shikimate pathway. The shikimate pathway is involved in the biosynthesis of aromatic amino acids, which are precursors for the synthesis of tocopherols. Yeast extract contains compounds such as phenolic acids, flavonoids and other secondary metabolites that can activate the shikimate pathway and stimulate the production of aromatic amino acids, leading to an increase in tocopherol biosynthesis. The application of CaONPs either individually or in synergism with yeast extract increased tocopherol production in the in vitro callus cultures of *S. chirata*. One possible mechanism for the increase in tocopherol contents by calcium oxide nanoparticles is through the activation of the antioxidant defense system in the callus cultures [34]. Nanoparticles can enter the cells and interact with the genetic material, leading to changes in gene expression and activation of antioxidant enzymes. For example, nanoparticles can upregulate the expression of genes involved in tocopherol biosynthesis, leading to increased tocopherol production. Additionally, studies [35,36] have shown that tocopherol biosynthesis is often induced by environmental stress, such as high light intensity or drought, and calcium oxide nanoparticles can mimic these stress conditions by regulating water availability and promoting antioxidant defense. Overall, the increase in tocopherol contents by calcium oxide nanoparticles in in vitro callus cultures is multifaceted and involves the activation of metabolic pathways, nutrient uptake and transport and environmental signaling.

Calcium oxide nanoparticles can increase the activities of phenylalanine ammonia-lyase (PAL), superoxide dismutase (SOD), catalase (CAT) and ascorbate peroxidase (APX) enzymes in plants. CAT and APX are antioxidant enzymes that protect cells from oxidative damage by scavenging hydrogen peroxide (H_2_O_2_). PAL is involved in the biosynthesis of phenolic compounds, including flavonoids and anthocyanins, while SOD is an antioxidant enzyme that protects cells from oxidative damage by scavenging superoxide radicals [37]. Nanoparticles can enter plant cells and interact with the genetic material, leading to changes in gene expression and enzyme activity. It might be assumed that application of CaONPs might have upregulated genes encoding antioxidant enzymes which resulted in enhanced production of these compounds. In addition, the nanoparticles may enhance the availability of nutrients or signaling molecules that are required for the biosynthesis or activation of PAL, SOD, CAT and APX. Calcium oxide nanoparticles can release calcium ions, which can act as secondary messengers in signaling pathways and regulate enzyme activity. The elicitation with yeast extracts in combination with CaONPs further boosted antioxidant production and functions [38].

One possible mechanism by which yeast extract can increase antioxidant activity is through the activation of signaling pathways involved in the biosynthesis of phenolic compounds. Yeast extract contains plant growth regulators such as auxins and cytokinin that can activate these pathways and stimulate the expression of antioxidant defense genes. This results in the production of these enzymes. Yeast extract is a rich source of amino acids, vitamins and minerals that are essential for plant growth and development. These nutrients can increase the expression of genes involved in the biosynthesis of antioxidant enzymes. The supply of nutrients might be taken as an assumed mechanism behind the increased antioxidant [39].

Elicitation boosted the phytochemical profile of the in vitro cultured *S. chirata*. Calcium oxide nanoparticles can influence the production of total flavonoids and anthocyanins in in vitro callus cultures. Flavonoids and phenolics are plant secondary metabolites with antioxidant, anti-inflammatory and other health-promoting properties. One possible mechanism for the influence of calcium oxide nanoparticles on flavonoid and phenolics production is through the activation of the phenylpropanoid pathway [40]. The phenylpropanoid pathway is responsible for the biosynthesis of a wide range of plant secondary metabolites, including flavonoids and anthocyanins. Calcium oxide nanoparticles can upregulate the activity of key enzymes involved in the phenylpropanoid pathway, such as phenylalanine ammonia-lyase (PAL), leading to increased production of flavonoids and anthocyanins [41]. The addition of yeast extract further improved the contents of total flavonoids and phenolics. Yeast extract contains compounds such as phenolic acids, flavonoids, and other secondary metabolites that can also activate the phenylpropanoid pathway in plant cells. The phenylpropanoid pathway is involved in the biosynthesis of phenolics and flavonoids. By activating this pathway, yeast extract can increase the production of these metabolites in callus cultures [42].

The application of CaONPs increased the DPPH scavenging activity from the *S. chirata* extracts. Calcium oxide nanoparticles are known to possess antioxidant properties, and DPPH (2,2-diphenyl-1-picrylhydrazyl) is a stable free radical that is commonly used to measure the antioxidant activity of compounds. When calcium oxide nanoparticles are applied to tissue culture, they may interact with the DPPH free radicals present in the culture. The nanoparticles may donate electrons to the free radicals, which neutralizes their reactivity and reduces their ability to damage cells. This process is known as scavenging, and it results in an increase in the percentage of DPPH scavenging [43].

Yeast extract contains various bioactive compounds such as amino acids, peptides and nucleotides that can act as antioxidants. These compounds can scavenge free radicals and reactive oxygen species, which contribute to oxidative stress and cell damage. Therefore, when yeast extract is added to the in vitro callus culture, it can increase the production of antioxidant compounds such as total phenolics, anthocyanins and flavonoids, which in turn, can enhance the DPPH scavenging percentage [44].

Yeast extracts contain various bioactive compounds such as amino acids, peptides and nucleotides that can act as elicitors to stimulate the production of secondary metabolites in plants [45]. In the case of Swertia species, previous studies have shown that yeast extracts can enhance the production of several bioactive compounds, including amarogentin, mangiferin and swertiamarin. In vitro callus cultures of Swertia can be used to produce these bioactive compounds under controlled conditions. When yeast extracts are added to the callus culture medium, they can stimulate the biosynthesis of secondary metabolites through the activation of various metabolic pathways. Yeast extracts can increase the activity of phenylalanine ammonia-lyase (PAL), which is a key enzyme involved in the production of phenolic compounds, including amarogentin, and mangiferin [46]. Moreover, yeast extracts can also enhance the activity of other enzymes involved in the biosynthesis of these compounds, such as UDP-glucosyltransferases and cytochrome P450 enzymes. These enzymes are responsible for the conversion of precursors into final products, such as mangiferin and swertiamarin. Similarly, the application of CaONPs boosts the production of these metabolites. This might be due to ROS signaling and the upregulation of biosynthetic genes involved in the production of these metabolites. For example, the upregulation of the gene encoding strictosidine synthase, a key enzyme in the biosynthesis of these compounds, can lead to an increase in their production. It might be assumed that application of CaONPs might have led to increased production of these metabolites through these molecular mechanisms [47,48].

## 4. Materials and Methods

### 4.1. Collection of Plant Material

Fresh shoots of *S. chirata* weighing 0.5 g were placed onto MS media in glass jars containing indole-3-butyric acid (IBA) at a concentration of 3 mg L^−1^ and kinetin (KN) at a concentration of 1 mg L^−1^. The media also contained 0.8% agar-agar, 3% *w*/*v* sucrose and was pH-adjusted to 5.6. The cultures were grown under controlled conditions, a temperature of 15 ± 1 °C and a humidity of approximately 74%. The photoperiod was set at 16 h of daylight and 8 h of darkness under aseptic conditions following [49].

### 4.2. Elicitation Experiments

Yeast Extract Powder (product Y-1625) was purchased from Sigma-Aldrich, St. Louis, MO, USA, with the appearance of a light beige to light brown, homogeneous powder. According to the supplier, it has a minimum total nitrogen content of 7.0% and a minimum amino nitrogen content of 1.5%. The loss on drying at 105 °C is not more than 5.0%, and the ash content is not more than 11.0%. The pH of a 2% aqueous solution is 7.0 ± 0.2, and it yields a clear to slightly hazy, yellow to brown solution. The clarity of the solution, as measured in NTU, is not more than 50. The insoluble matter is not more than 0.2%, and the endotoxin level is not more than 0.05 EU/mL. Additionally, the product conforms to microbiological performance testing.

Sigma-Aldrich provided calcium oxide nanoparticles that were 99% pure and had a particle size of less than 100 nm, as determined by transmission electron microscopy (TEM). According to the supplier, the nanoparticles have a specific surface area of 30–40 m^2^/g and a density of 3.3 g/cm^3^. They appear white in color and have a chemical formula of CaO with a CAS number of 1305-78-8.

**Figure 3:**
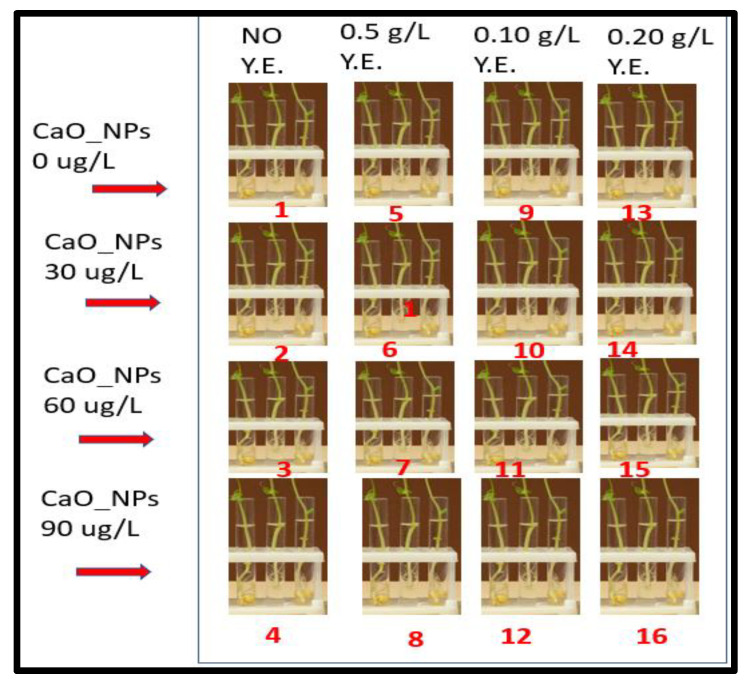
A graphical representation of the experimental design. A total of 48 test tubes with 16 elicitation treatments (represented with numbers in red fonts) and 3 replicates containing tissue cultures were arranged. The treatments differed in rows and columns. Interaction of treatments in rows and columns was studied along with individual effects of the treatments on the in vitro cultures of *Swertia chirata*.

To prepare for the culture experiment, calli of *Swertia chirata* were cut into small pieces after 34 days and placed on sterile filter paper. These pieces were then cultured on MS media, and three treatment regimens of CaONPs as elicitors (30 μg/L, 60 μg/L and 90 μg/L) [50] and three treatment concentrations of yeast extract (0.5, 0.10 and 0.20 g/L) were chosen. A control treatment consisting of MS media alone without any elicitor treatment was also included for comparison (treatment 1). The experiment involved 48 autoclaved test tubes, each containing approximately 9 mL of medium and sealed with a cotton plug. See Figure 3 for the graphical design of the experiment. The first column consists of 12 test tubes with four treatments, each having three replicates. These test tubes were subjected to no elicitation treatment (T_1_) and elicitation with 30, 60 and 90 μg/L CaONPs alone (T_2_, T_3_ and T_4_, respectively). The second column contained 12 test tubes with the next 4 treatments, each having 3 replicates. These test tubes were elicited with 0.5 g/L yeast extract and 0 ug/L CaONPs, as well as 0.5 g/L yeast extract and one of three concentrations of CaONPs (30, 60 and 90 μg/L) and designated as T_5_, T_6_, T_7_ and T_8_, respectively. The third column contains twelve test tubes with the next four treatments, each having three replicates. These test tubes were elicited with 0.10 g/L yeast extract and 0 μg/L CaONPs, as well as 0.10 g/L yeast extract and one of three concentrations of CaONPs (30, 60, and 90 μg/L) and designated as T_9_, T_10_, T_11_ and T_12_, respectively. Finally, the fourth column consisted of twelve test tubes with the next four treatments, each having three replicates. These test tubes were elicited with 0.20 g/L yeast extract and 0 μg/L CaONPs, as well as 0.20 g/L yeast extract and one of three concentrations of CaONPs (30, 60 and 90 μg/L) and designated as T_13_, T_14_, T_15_ and T_16_, respectively. Interaction of treatments in rows and columns was studied along with individual effects of the treatments on the in vitro cultures of *Swertia chirata* [51].

The cultures were incubated at 25 °C with a 16/8 h photoperiod in the growth chamber to promote callus proliferation. After 50 days, data on fresh weight (FW), dry weight (DW), callus moisture content and callus morphological traits were collected.

To determine the FW, calli extracted from each treatment were cleaned with sterile distilled water, placed on filter paper, squeezed with forceps to drain excess water and weighed. Each callus was then dried in an oven (50 °C) for 24 h before being weighed again. The FW and DW data were converted to g/l. The percentage moisture content in CC was calculated using the FW and DW of the callus with the formula provided by Rashmi and Trivedi [52]:

Percentage moisture contents = (B − A) − (C − A)/(B − A) × 100

where A = weight of empty petri dish; B = weight of petri dish with fresh callus; and C = weight of petri dish with dried callus.

Observations were recorded on callus nature, response and color as well to understand the impacts of elicitation on callus morphological health.

### 4.3. Extract Preparation

The matured shoots were taken out, dried and then crushed into a fine powder with the help of liquid nitrogen. This powder was then mixed with 100 mL of 80% methanol and kept for a whole night. The next day, the mixture was sonicated for 10 min at 30% amplitude with 2 s of pulse using SONICS Vibra Cell^TM^. After sonication, the mixture was centrifuged at 10,000 rpm for 15 min at 4C using Eppendorf of 5804 R. The resulting liquid on top was filtered using syringe filters with a pore size of 0.22 μm (PVDF). The remaining mixture was stored at 4C for future analysis following Gupta and Sood [49].

### 4.4. Quantification of Amarogentin and Mangiferin

Shoot cultures cultivated under different elicitor treatments were analyzed using reverse-phase high-performance liquid chromatography (HPLC) with the assistance of an HPLC pump and a C18 (5 μm) Waters column. A Photodiode Array Detector (Waters 2996) was employed to determine the quantities of amarogentin and mangiferin. The filtrate was diluted by a factor of 10 and injected into the column. Solvent A, consisting of 0.1% trifluoroacetic acid (Merck, Rahway, NJ, USA), and Solvent B, a solvent system comprising 70% acetonitrile and 30% water (Merck:MilliQ), were used. The column was eluted at a flow rate of 1.0 mL/min in an isocratic mode, and amarogentin and mangiferin were detected at 270 nm. The column temperature was maintained at 25 degrees Celsius, and the entire cycle lasted for 30 min. The quantification of the bioactive compounds (amarogentin and mangiferin) in the plant extracts was performed by comparing their retention times with the authentic standards obtained from Chromadex, Inc. in India. The results were reported as micrograms per milligram of dry weight (DW) [49].

### 4.5. Determination of Total Phenolic Content

The total phenolic content (TPC) of *S. chirata* plant extracts, was determined using the method described by Kim et al. [53], with a few modifications. In this method, 0.1 mL of plant extract was mixed with 0.4 mL of distilled water, followed by the addition of 0.15 mL of Folin–Ciocalteu reagent. After thorough mixing, the mixture was left at room temperature for 5 min. Subsequently, 0.5 mL of 20% Na_2_CO_3_ was added, and the mixture was incubated in a dark room for 1 h. The absorbance was measured at 750 nm using a UV–visible spectrophotometer. To plot the calibration curve, gallic acid (100–500 micrograms per milliliter) was used. The total phenolic content was determined using the calibration curve equation (y = 0.003x − 0.046, r^2^ = 0.996), and the results were reported as milligrams of gallic acid equivalent per gram of the extract (mg GA g^−1^).

### 4.6. Determination of Total Flavonoid Content

The total flavonoid content (TFC) of *S. chirata* plant extracts, was determined using a modified version of the method developed by Ebrahimzadeh et al. [54]. In this procedure, 0.1 mL of the plant extract in methanol was mixed with 0.4 mL of methanol. Then, 0.1 mL of 10% AlCl_2_ was added, followed by the addition of 0.1 mL of 1 M sodium acetate to make the final volume up to 4 mL with distilled water. The mixture was then incubated at room temperature for 30 min. The absorbance was measured at 415 nm using a UV–visible spectrophotometer. Quercetin (100–500 micrograms per milliliter; Sigma-Aldrich, Bangalore, India) was used as a standard to plot the calibration curve. The flavonoid content was calculated using the calibration curve equation (y = 0.001x − 0.019, r^2^ = 0.993), and the total amount of flavonoids was expressed as milligrams of quercetin equivalent per gram of the extract (mg QE g^−1^).

### 4.7. Determination of DPPH Scavenging Percentage

The antioxidant activity of *S. chirata* shoot culture extracts, was assessed using the DPPH free radical scavenging assay based on the method described by Yesmin et al. [55], with some modifications. In this method, 50 microliters of the plant extract was mixed with 3 mL of methanolic DPPH solution (0.004%). The mixture was then kept in the dark at room temperature for 30 min. The absorbance was measured at 517 nm using a UV–visible spectrophotometer. The free radical scavenging activity (RSA) of the different extracts was compared to that of BHT (Sigma-Aldrich) used as a standard. The DPPH free radical scavenging activity (%RSA) was calculated using the following formula:

%RSA = {(Absorbance of control − Absorbance of Sample)/Absorbance of control} × 100

### 4.8. Determination of Activities of Bioactive Antioxidants

To assess the antioxidant enzyme activity of specific callus samples, a modified version of the Khan et al. [56] method was used. Each sample was mixed with 2 mL of phosphate buffer (50 mM, 1% PVP, 0.1 mM EDTA, pH 7.8) and homogenized to create a uniform mixture. The samples were then subjected to two rounds of centrifugation at 4 °C and 12,000 rpm for a total of 15 min to produce a uniform reaction buffer. The supernatants obtained from each sample were collected and utilized to measure the activity of antioxidant enzymes including superoxide dismutase (SOD; EC 1.15.1.1), catalase (CAT; EC 1.11.1.6), and peroxidase (POD; EC 1.11.1.7), by following the methods described by Giannopolitis and Ries [57], Arrigoni et al. [58] and Abeles and Biles [59], respectively. Furthermore, for the phenylalanine ammonia lyase (PAL) assay [56], 100 mg of each sample was homogenized in chilled potassium borate buffer (concentration = 100 mM, pH = 8.8, with 2 mM mercaptoethanol). The mixture was then subjected to centrifugation at 4 °C and 12,000 rpm for 10 min. The reaction mixture was adjusted by adding 0.5 mL of the extract from each chosen sample to a tube containing 1.0 mL of potassium borate buffer and 0.5 mL of phenylalanine (concentration = 4.0 mM, pH = 8.8). After 30 min, 2.0 mL of the reaction mixture was combined with 0.2 mL of 6 M HCl. The absorbance at 290 nm was measured after 30 min to determine the enzyme product formed, taking into account the absorbance of the reaction mixture. One unit of phenylalanine ammonia lyase activity (U) was defined as a 0.01 absorbance fluctuation.

### 4.9. Determination of Anthocyanin and Tocopherol Contents

The callus was first dried using a paper towel and then frozen. The pigments were extracted using the method described by [60] and subjected to chromatography using BAW 4:1:5, Bu-HCI 1:1 and 1% HCI as per the procedure outlined by [61]. The absorbance of anthocyanin was measured at 525 nm wavelength. The content of alpha tocopherols was evaluated following Baker [62].

### 4.10. Statistical Analysis and Experimental Layout

All experiments were conducted using a completely randomized design. Each treatment was replicated three times, and the data were analyzed using a one-way analysis of variance in Costat software version 6.3 (developed by Cohort software, Berkeley, CA, USA). The mean values and statistically significant differences (*p* < 0.05) were determined using DMRT [50].

## 5. Conclusions

In this study, we conducted an investigation into the effects of yeast extract and calcium oxide nanoparticles (CaONPs) elicitation on in vitro cultures of *Swertia chirata*. Our research focused on assessing the impact of different concentrations of CaONPs in combination with varying concentrations of yeast extract on callus growth, antioxidant activity and phytochemical contents. The results demonstrated that the elicitation with Y.E. and CaONPs significantly influenced the growth and characteristics of *S. chirata* callus cultures. Specifically, the treatments involving yeast extract and CaONPs exhibited the most notable effects, leading to increased levels of total flavonoids, total phenolics, amarogentin and mangiferin. These treatments also resulted in improvements in total anthocyanin and alpha tocopherol contents, as well as enhanced DPPH scavenging activity. Additionally, the elicitation treatments significantly improved callus growth and characteristics, including an elevation from an average to an excellent level of callus response, color transformation from yellow to yellow-brown and greenish, and texture enhancement from fragile to compact. The most favorable response was observed in treatments involving 0.20 g/L yeast extract and 90 µg/L CaONPs. Overall, our findings suggest that the elicitation strategy employing yeast extract and CaONPs holds promise in promoting the growth, antioxidant activity and phytochemical contents of *S. chirata* callus cultures. These findings have important implications for the development of efficient protocols for plant tissue culture and the production of phytochemicals with potential health benefits.

## Figures and Tables

**Table 1:** Morphological attributes of callus cultures of *S. chirata* as affected by different concentrations of CaONPs and yeast extracts.

Treatments	Callus Nature	Callus Response ^a^	Callus Color
T_1_	Fragile	++	Yellow
T_2_	Fragile	++	Yellow-brown
T_3_	Fragile	++	Yellow-brown
T_4_	Fragile	++	Yellow-brown
T_5_	Fragile	+++	Yellow-brown
T_6_	Semi Compact	++++	Yellow greenish
T_7_	Compact	+++++	Greenish
T_8_	Compact	+++++	Greenish
T_9_	Fragile	++	Yellow-brown
T_10_	Semi Compact	++++	Yellow greenish
T_11_	Compact	+++++	Greenish
T_12_	Compact	+++++	Greenish
T_13_	Fragile	++	Yellow-brown
T_14_	Compact	+++++	Greenish
T_15_	Compact	+++++	Greenish
T_16_	Compact	+++++	Greenish

^a^ +++++ = excellent; ++++ = very good; +++ = average; ++ = poor.

**Table 2:** LSD test interpreted mean values of growth and biomass attributes of callus cultures of *S. chirata* as affected by different concentrations of CaONPs and yeast extracts.

Treatments	Shoot Length (cm)		Root Length (cm)		No. of Shoots/Explant		No. of Roots per Shoots	
	Mean	±S.D.	Mean	±S.D.	Mean	±S.D.	Mean	±S.D.
T_1_	2.59 m	0.020	0.99 m	0.025	2.17 l	0.286	1.34 k	0.040
T_2_	2.66 kl	0.017	1.10 l	0.015	2.60 k	0.173	1.43 j	0.025
T_3_	2.73 k	0.021	1.24 k	0.035	2.97 j	0.058	1.46 ij	0.006
T_4_	2.95 j	0.040	1.38 j	0.036	3.23 i	0.252	1.49 lk	0.012
T_5_	2.64 lm	0.025	1.13 l	0.015	2.30 l	0.100	1.35 k	0.026
T_6_	3.10 i	0.032	1.51 i	0.045	3.75 g	0.145	1.52 h	0.020
T_7_	3.24 h	0.051	1.67 h	0.035	4.02 f	0.076	1.58 g	0.021
T_8_	3.51 f	0.065	1.77 g	0.026	4.23 f	0.076	1.64 f	0.015
T_9_	2.72 kl	0.025	1.30 k	0.015	2.93 j	0.058	1.47 i	0.021
T_10_	3.34 g	0.031	1.95 f	0.045	4.48 e	0.126	1.69 e	0.015
T_11_	3.65 e	0.095	2.12 e	0.035	4.83 d	0.070	1.76 d	0.015
T_12_	4.06 d	0.075	2.25 d	0.035	5.12 c	0.104	1.82 c	0.025
T_13_	2.91 i	0.040	1.42 j	0.035	3.47 h	0.058	1.51 h	0.017
T_14_	4.43 c	0.072	2.42 c	0.042	5.47 b	0.153	1.91 b	0.021
T_15_	4.71 b	0.045	2.61 b	0.035	5.75 a	0.050	1.96 a	0.015
T_16_	5.07 a	0.062	2.79 a	0.021	5.95 a	0.050	2.00 a	0.015
LSD = 0.05	0.083		0.054		0.222		0.034	

Means following a different alphabet differ significantly from each other at LSD 5%.

**Table 3:** DPPH scavenging activity, callus moisture contents and some biochemical markers of callus cultures of *S. chirata* as affected by different concentrations of CaONPs and yeast extracts.

Treatments	Total Anthocyanin (mg/100 g FW)		DPPH Scavenging Percentage		Alpha Tocopherol (μg/g FW)		Callus Moisture Content (%)	
	Mean	±S.D.	Mean	±S.D.	Mean	±S.D.	Mean	±S.D.
T_1_	4.35 m	0.040	28.67 m	2.082	1.67 n	0.060	29.67 n	1.528
T_2_	4.45 l	0.025	36.33 l	2.517	1.86 m	0.050	35.0 m	1.732
T_3_	4.66 k	0.026	42.67 k	1.528	1.95 l	0.026	40.33 l	1.155
T_4_	4.76 j	0.025	46.33 j	1.528	1.98 kl	0.020	43.67 jk	1.155
T_5_	4.48 l	0.045	34.33 l	0.577	2.07 ij	0.020	33.6 m	2.082
T_6_	4.93 i	0.040	51.33 i	1.528	2.14 h	0.035	46.33 ij	1.528
T_7_	5.07 h	0.049	55.67 h	1.155	2.24 g	0.021	51.67 h	2.082
T_8_	5.24 g	0.070	59.33 g	1.528	2.39 f	0.045	55.00 g	1.000
T_9_	4.70 jk	0.067	46.67 j	1.528	2.04 jk	0.045	42.3 kl	1.155
T_10_	5.62 f	0.070	64.33 f	2.517	2.43 f	0.075	59.33 f	1.528
T_11_	5.88 e	0.112	70.33 e	2.517	2.56 e	0.031	63.33 e	2.082
T_12_	6.09 d	0.081	76.33 d	1.528	2.68 d	0.031	67.00 d	1.000
T_13_	4.77 j	0.032	48.33 ij	1.528	2.13 hi	0.025	48.00 i	1.732
T_14_	6.82 c	0.035	80.33 c	1.155	2.93 c	0.056	74.33 c	2.887
T_15_	7.05 b	0.051	85.00 b	2.000	3.01 b	0.026	78.33 b	0.577
T_16_	7.19 a	0.045	88.67 a	2.517	3.19 a	0.070	83.67 a	3.055
LSD = 0.05	0.093		3.026		0.072		2.940	

FW: Fresh weight; S.D. standard deviation. Means following a different alphabet differ significantly from each other at LSD 5%.

**Table 4:** Phytochemical attributes of callus cultures of *S. chirata* as affected by different concentrations of CaONPs and yeast extracts.

Treatments	Mangiferin		Amarogentin		Total Flavonoids		Total Phenolics	
	Mean	±S.D.	Mean	±S.D.	Mean	±S.D.	Mean	±S.D.
T_1_	9.12 n	0.023	4.37 m	0.062	12.96 p	0.059	8.20 o	0.075
T_2_	9.35 m	0.040	4.54 l	0.025	14.15 n	0.133	9.12 m	0.125
T_3_	9.53 l	0.021	4.74 k	0.025	15.00 m	0.105	9.99 l	0.100
T_4_	9.67 jk	0.025	4.85 j	0.030	16.43 j	0.086	11.35 j	0.120
T_5_	9.38 m	0.025	4.44 lm	0.015	13.33 o	0.111	8.69 n	0.071
T_6_	10.29 i	0.053	5.03 i	0.057	17.32 i	0.086	12.42 i	0.101
T_7_	10.81 h	0.055	5.24 h	0.030	18.69 h	0.060	13.67 h	0.105
T_8_	11.30 g	0.062	5.38 g	0.045	19.91 g	0.062	14.20 g	0.080
T_9_	9.58 kl	0.070	4.75 jk	0.025	15.16 l	0.061	10.11 l	0.120
T_10_	11.67 f	0.121	5.59 f	0.085	20.42 f	0.103	16.43 f	0.086
T_11_	11.96 e	0.047	6.12 e	0.115	21.48 e	0.135	17.16 e	0.050
T_12_	12.42 d	0.163	6.52 d	0.070	23.54 d	0.081	18.11 d	0.105
T_13_	9.71 j	0.050	4.84 jk	0.046	16.07 k	0.115	11.04 k	0.072
T_14_	13.34 c	0.110	6.80 c	0.075	25.58 c	0.108	18.43 c	0.086
T_15_	14.29 b	0.052	7.12 b	0.116	27.15 b	0.078	19.39 b	0.046
T_16_	15.07 a	0.062	7.46 a	0.060	28.86 a	0.146	20.17 a	0.074
LSD = 0.05	0.119		0.104		0.165		0.152	

Means following a different alphabet differ significantly from each other at LSD 5%.

**Table 5:** Antioxidant metabolites of callus cultures of *S. chirata* as affected by different concentrations of CaONPs and yeast extracts.

Treatments	PAL (U/g FW)		SOD (U/g FW)		POD (U/g FW)		CAT (U/g FW)	
	Mean	±S.D.	Mean	±S.D.	Mean	±S.D.	Mean	±S.D.
T_1_	1.83 k	0.023	0.70 j	0.006	0.67 l	0.006	0.19 j	0.005
T_2_	1.99 ij	0.016	0.71 j	0.003	0.70 kl	0.002	0.21 ij	0.004
T_3_	2.09 i	0.069	0.73 j	0.004	0.71 jkl	0.004	0.23 ij	0.006
T_4_	2.19 h	0.022	0.77 ij	0.006	0.72 jkl	0.003	0.26 hij	0.004
T_5_	2.08 i	0.025	0.82 i	0.009	0.76 ijk	0.004	0.30 hi	0.003
T_6_	2.23 h	0.085	1.03 h	0.091	0.80 i	0.010	0.33 h	0.008
T_7_	3.10 g	0.086	1.49 g	0.076	1.81 g	0.037	0.67 g	0.016
T_8_	3.37 f	0.072	3.28 d	0.056	1.95 f	0.057	1.99 e	0.091
T_9_	1.90 jk	0.031	0.76 ij	0.023	0.71 jkl	0.004	0.24 ij	0.003
T_10_	3.44 f	0.111	1.75 f	0.071	1.30 h	0.066	0.81 f	0.022
T_11_	3.94 e	0.051	4.07 b	0.061	2.96 d	0.047	2.17 d	0.166
T_12_	4.14 d	0.078	2.15 e	0.061	2.53 e	0.081	1.99 e	0.025
T_13_	2.07 i	0.053	0.78 ij	0.003	0.77 ij	0.006	0.26 ij	0.008
T_14_	5.16 c	0.045	3.96 c	0.057	3.21 c	0.075	2.45 c	0.056
T_15_	5.37 b	0.026	4.12 b	0.040	3.40 b	0.031	2.69 b	0.034
T_16_	5.53 a	0.021	4.34 a	0.098	3.68 a	0.031	2.84 a	0.022
LSD = 0.05	0.096		0.088		0.066		0.085	

Means following a different alphabet differ significantly from each other at LSD 5%.

**Table 6:** Spearman correlation matrix for the studied variables of the *S. chirata*.

Variables	PAL	SOD	POD	CAT	CMC	Mang	Amaro	TF	TP	Antho	DPPH	SL	RL	Nos/e	Nor/s
PAL	1														
SOD	0.942 *														
POD	0.954 *	0.984 *													
CAT	0.961 *	0.984 *	0.987 *												
CMC	0.950 *	0.926 *	0.956 *	0.937 *											
Mang	0.966 *	0.943 *	0.965 *	0.952 *	0.992 *										
Amaro	0.964 *	0.930 *	0.952 *	0.937 *	0.990 *	0.995 *									
TF	0.963 *	0.928 *	0.950 *	0.941 *	0.989 *	0.992 *	0.996 *								
TP	0.964 *	0.928 *	0.950 *	0.941 *	0.987 *	0.993 *	0.996 *	0.998 *							
Antho	0.961 *	0.941 *	0.962 *	0.952 *	0.989 *	0.995 *	0.993 *	0.991 *	0.991 *						
DPPH	0.945 *	0.934 *	0.955 *	0.941 *	0.987 *	0.991 *	0.992 *	0.989 *	0.989 *	0.993 *					
SL	0.967 *	0.938 *	0.959 *	0.946 *	0.982 *	0.991 *	0.993 *	0.991 *	0.992 *	0.988 *	0.986 *				
RL	0.964 *	0.947 *	0.967 *	0.953 *	0.992 *	0.998 *	0.995 *	0.993 *	0.992 *	0.996 *	0.992 *	0.990 *			
Nos/e	0.958 *	0.922 *	0.948 *	0.933 *	0.990 *	0.990 *	0.993 *	0.992 *	0.993 *	0.989 *	0.988 *	0.990 *	0.989 *		
Nor/s	0.947 *	0.921 *	0.949 *	0.930 *	0.992 *	0.991 *	0.994 *	0.989 *	0.988 *	0.990 *	0.989 *	0.984 *	0.989 *	0.987 *	
Toco	0.939 *	0.965 *	0.979 *	0.967 *	0.968 *	0.970 *	0.956 *	0.956 *	0.954 *	0.965 *	0.966 *	0.954 *	0.975 *	0.955 *	0.954 *

* Significantly differ from zero at alpha 0.05. PAL: Phenylalanine ammonia-lyase; SOD: Superoxide dismutase; Amaro: Amarogentin; SL: Shoot length; RL: Root length; Toco: Tocopherols; Nor/s: Number of roots/shoot; Nos/e: Number of shoots per explant; Antho: Anthocyanin; TF: Total phenolics; CAT: Catalase; Mang: Mangiferin; CMC: Callus moisture contents; TP: Total phenolics; POD: Peroxidase; DPPH: 2,2-diphenyl-1-picrylhydrazyl.

**Table 7:** Two-way analysis of variance (ANOVA) results as affected by various elicitation concentrations of CaONPs and Yeast extracts.

Variation Source	^a^ *df*	Shoot Length	Root Length	No. of Shoots/Explant	No. of Roots/Shoots	Callus Moisture	DPPH Scavenging %
Treatment effect in rows (F1)	3	2.997 ^b^ *** (0.000)	1.623 *** (0.000)	8.781 *** (0.000)	0.234 *** (0.000)	1317.90 *** (0.000)	1849.13 *** (0.000)
Treatment effect in columns (F2)	3	5.197 *** (0.000)	2.857 *** (0.000)	12.848 *** (0.000)	0.401 *** (0.000)	2570.40 *** (0.000)	3156.54 *** (0.000)
Interaction (F1 × F2)	9	0.378 *** (0.000)	0.121 *** (0.000)	0.305 *** (0.000)	0.014 *** (0.000)	56.61 *** (0.000)	71.48 *** (0.000)
Error	32	0.002	0.001	0.017	0.00004	3.125	3.312
**Variation Source**	***df***	**Anthocyanin**	**Tocopherol**	**Superoxide dismutase**	**Catalase**	**Peroxidase**	**Phenylalanine ammonia lyase**
Treatment effect in rows (F1)	3	3.691 *** (0.000)	0.757 *** (0.000)	9.233 *** (0.000)	5.254 *** (0.000)	6.071 *** (0.000)	8.190 *** (0.000)
Treatment effect in columns (F2)	3	8.312 *** (0.000)	1.896 *** (0.000)	13.823 *** (0.000)	7.203 *** (0.000)	9.175 *** (0.000)	13.716 *** (0.000)
Interaction among the factors (F1 × F2)	9	0.565 *** (0.000)	0.084 *** (0.000)	2.948 *** (0.000)	1.213 *** (0.000)	1.281 *** (0.000)	1.441 *** (0.000)
Error	32	0.003	0.001	0.002	0.002	0.001	0.003
**Variation Source**	***df***	**Amarogentin**	**Mangiferin**	**Flavonoids**	**Phenolics**		
Treatment effect in rows (F1)	3	4.810 *** (0.000)	16.942 *** (0.000)	136.33 *** (0.000)	98.598 *** (0.000)		
Treatment effect in columns (F2)	3	8.650 *** (0.000)	29.668 *** (0.000)	209.283 *** (0.000)	138.373 *** (0.000)		
Interaction among the factors (F1 × F2)	9	0.57 *** (0.000)	2.5001 *** (0.000)	10.783 *** (0.000)	5.966 *** (0.000)		
Error	32	0.003	0.005	0.009	0.008		

^a^ *df*. Degree of freedom ^b^ *** = significant at 0.001 level.

## Data Availability

All data are available within this publication.
